# On-Demand Porous Carbon Fabrication via Selective Laser Sintering for Electrochemical Energy Storage

**DOI:** 10.1021/acsaenm.5c00297

**Published:** 2025-08-07

**Authors:** Anthony Griffin, Muxuan Yang, Parker Frame, Weinan Xu, Zhe Qiang

**Affiliations:** † School of Polymer Science and Engineering, 1076 University of Southern Mississippi, Hattiesburg, Mississippi 39406, United States; ‡ School of Polymer Science and Polymer Engineering, The University of Akron, Akron, Ohio 44325, United States

**Keywords:** 3D-printing, carbon−metal nanocomposites, polyolefin, supercapacitors, pyrolysis, hierarchical porosity

## Abstract

Manufacturing structured carbon with tunable three-dimensional (3D) architectures remains a major hurdle for widescale use due to process complexity and high cost of current methods. This work demonstrates the fabrication of structured carbon using selective laser sintering (SLS)-based additive manufacturing, enabling control over both the macroscopic geometry and the nanoscale pore textures. Our process employs polyethylene (PE) as the carbon precursor and only involves steps of printing, cross-linking, and pyrolysis. The incomplete coalescence of PE particles during printing results in the formation of a macroporous structure. Moreover, we demonstrate the production of 3D-printed carbon–cobalt nanocomposites through a simple metal immersion step prior to pyrolysis. The electrochemical properties of these structured carbons and carbon–cobalt nanocomposites were investigated, revealing enhanced performance attributed to the synergistic effects of electric double-layer capacitance and pseudocapacitance. Our method is resource-efficient, utilizes inexpensive precursors, and is capable of imparting functional nanoparticles to the carbon matrix. The resulting structured carbon-based electrodes exhibit high charge storage capacity, highlighting their potential for next-generation, 3D-printable electrochemical energy storage devices.

## Graphical abstract

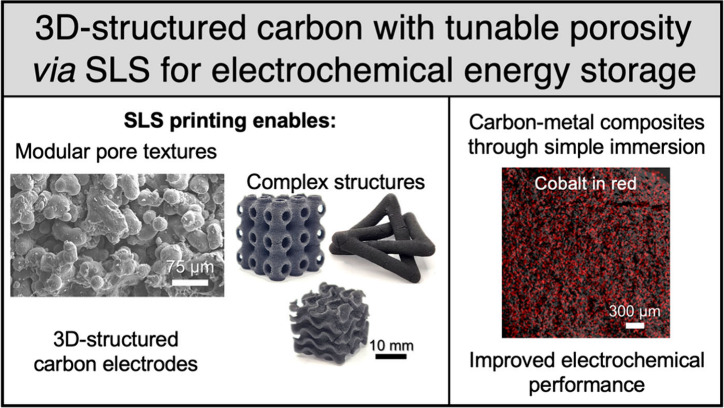

## 1 Introduction

While carbon materials have played vital roles in various applications, including thermal management,^1–4^ energy storage,^5–7^ high-performance composites,^8,9^ and water remediation,^10–12^ their use is often limited in the form of additives or fillers due to the lack of ability to control their macroscopic structure for direct use.^13–15^ In turn, most functional carbons including graphene,^16,17^ carbon nanotubes,^18,19^ and quantum dots^20,21^ are incorporated and dispersed in a matrix to enable their processing into various composite-based products. Noteworthily, with the advent of additive manufacturing (AM), significant efforts have been made to produce macroscopic carbon structures on-demand.^22,23^ Coupling the vast geometric freedom that 3D-printing confers with high resource efficiency, AM is anticipated to expand into several industrial sectors and facilitate significant technological development.^24–26^ In general, the production of three-dimensionally (3D)-structured carbons has been achieved through two main approaches: the use of carbon-containing suspensions coupled with postprocessing steps and preparing polymeric carbon precursors followed by pyrolysis. Specifically, aqueous carbon inks have been employed to yield 3D-structured carbon following a freeze-drying process.^27^ Alternatively, polymeric resins that can serve as carbon precursors have been demonstrated in several systems, including fused filament fabrication (FFF), direct laser writing (DLW), and vat photopolymerization (VP) printing followed by pyrolysis to prepare monolithic carbons.^28–31^ While these strategies are capable of forming structured carbons with improved mechanical properties, they typically exhibit notable dimensional shrinkage (typically ∼60% or higher) as well as rely on using relatively expensive chemicals as starting materials.^32,33^ It is worth noting that there is a recent study which has shown that relatively inexpensive VP resins can be utilized to prepare hierarchically porous, 3D-structured carbon lattices.^34^

Recent studies have demonstrated the use of polyolefins, such as polypropylene (PP), as precursors in conjunction with fused filament fabrication (FFF) printing for the production of structured carbons.^35,36^ In this system, a crack-facilitated diffusion mechanism of sulfonation-induced cross-linking was observed, attributed to the significantly large critical dimension of FFF-printed layers (at least 0.4 mm). Following pyrolysis, cross-linked parts were converted to carbon with the retention of complex architectures and controlled dimensional shrinkage. However, there are several limitations in this method, including inevitable microcrack formation, limited printing resolution and speed, and anisotropic shrinkage behaviors. It is also worth highlighting that AM of polyacrylonitrile (PAN), which could be utilized as a carbon precursor, has been achieved through VP using an interfacial photopolymerization process where rudimentary multilayer shapes were demonstrated.^37^ However, VP of semicrystalline polyolefins for direct part fabrication remains a challenge. Alternatively, selective laser sintering (SLS) is a highly promising AM strategy that uses a laser to selectively melt and fuse polymeric particles layer by layer until a 3D object is completed.^38,39^ Through this strategy, structures have been prepared with tunable hierarchical porosity dependent on the degree of particle coalescence.^40^ Furthermore, SLS, in comparison to FFF printing, can reach higher print resolutions (down to ∼70–100 μm) as well as eliminate the need of support structures.^41–43^ It is worth noting that SLS printing of polyethylene (PE) has been historically challenging due to its irregular particle morphologies, narrow processing windows, and possible polymer degradation. However, coupling a temperature-induced phase separation (TIPS) strategy with small loadings of carbon black can enable its SLS printability.^40^ Due to the advantages offered by SLS, this 3D-printing strategy has been employed to fabricate complex structures for many applications, including scalable and precise electrode manufacturing.^44,45^ However, in these studies, the high content of electrochemically inactive polymer matrix in the final electrode has limited composite performance.^46,47^ It should be noted that 3D-structured ceramic electrodes have recently been demonstrated in several energy storage applications.^48–50^ However, the direct fabrication of fully active carbon electrode materials with controlled 3D structures is lacking, and addressing this gap could enable advancement in producing high-performance structured electrodes.

This work demonstrates the fabrication of 3D-structured carbons with hierarchical porosity and high printing resolution from PE precursors by using SLS printing. Following a two-step process of cross-linking and pyrolysis, complex carbon structures can be prepared on-demand with relatively low isotropic dimensional shrinkage (between 8 and 11%) compared to initial printed specimens. Additionally, adjusting the cross-linking reaction conditions enables further control over particle coalescence, allowing for precise tuning of the resulting carbon porosity. In comparison to structured carbons prepared through alternate AM processes, such as FFF printing, this SLS-derived method can prepare carbon monoliths with higher print resolution and nearly isotropic shrinkage behavior, without the need for support structures, in addition to modular pore textures controlled through printing parameters. A simple metal infiltration step was introduced prior to pyrolysis to prepare carbon–metal nanocomposites with well-dispersed cobalt nanoparticles. These structured carbons and carbon–cobalt nanocomposites were then employed as electrodes for electrochemical energy storage. It was found that the carbon–cobalt nanocomposite exhibited improved electrochemical performance due to synergistic effects from electric double-layer capacitance and pseudocapacitance.

## 2 Experimental Section

### 2.1 Materials Preparation

PE microparticles for SLS printing were prepared from a 15 wt % solution of low-density PE (number-averaged molecular weight, or Mn: 36 300 g/mol, Sigma-Aldrich) in *o*-xylene that was held under reflux conditions (∼140 °C) for 2 h followed by cooling rapidly in an ice bath down to 10 °C.^40^ Precipitated microparticles were obtained through vacuum-assisted filtration, which were then washed with ethanol (Sigma-Aldrich) three times, vacuum-dried at 75 °C overnight, and sieved with a 100-mesh sieve (149 μm pore size). To enhance optical adsorption of the PE microparticles, 5 wt % carbon black (VULCAN XC72R) was mixed with the PE microparticles using a high-speed analytical mill. The carbon black has a particle size reported to be 20–50 nm with a surface area of approximately 241 m^2^/g. SLS 3D-printing was carried out with a Sintratec Kit desktop SLS printer equipped with a 445 nm diode laser with 2.3 W of power. To briefly describe the printing conditions, a laser speed of 300 mm/s, preheating temperature of 85 °C, and printing temperature of 105 °C were used. Detailed printing procedures can be found in a previous report.^40^ While a cube structure (1 cm height, width, and length) was used as a model system and for understanding the dimensional shrinkage of samples from our process, several complex structures were also investigated in this study.

SLS-printed PE parts were fully submerged in concentrated sulfuric acid (98%, purchased from Fisher Scientific) and heated to 120 °C for varying amounts of time in a Thermolyne F6010 muffle furnace (Thermo Scientific). Cross-linked samples were then allowed to cool to room temperature, removed from reaction vessels, and washed three times with deionized water (Millipore Sigma Milli-Q IQ 7003) to remove reaction byproducts and residual acids. The washed materials were then dried overnight under a vacuum at 55 °C and carbonized under a nitrogen atmosphere with a tube furnace (Across international TF1400) heating to 600 °C with a ramp rate of 1 °C/min followed by heating to 800 °C/min with a rate of 5 °C/min. To prepare carbon–cobalt nanocomposites, dried cross-linked structures were submerged in 50:50 ethanol–DI water volumetric mixtures containing cobalt­(II) nitrate hexahydrate (Thermo Scientific) at various molar concentrations (between 0.5 and 5 M). Following 1 h of soaking time at 60 °C and subsequent drying at 100 °C overnight, cobalt loaded samples were then pyrolyzed under nitrogen by heating to 600 °C with a ramp rate of 1 °C/min, followed by heating to 1300 °C with a rate of 5 °C/min and then held for 3 h.

### 2.2 Characterization

Scanning electron microscopy (SEM) was performed using a Zeiss Ultra 60 field emission microscope with an accelerating voltage of 11 kV to characterize the morphology of printed structures coupled with energy-dispersive X-ray spectroscopy (EDS) to assess elemental compositions. Differential scanning calorimetry (DSC) was performed with a Discovery 250 instrument (TA Instruments) to determine the degree of crystallinity of PE structures by comparing the second melting peak to the theoretical PE melting enthalpy (293 J/g).^51^ Samples were first heated from 25 to 250 °C with a rate of 10 °C/min in order to erase thermal history, followed by cooling to 25 °C with a rate of 5 °C/min and heating to 250 °C with a rate of 10 °C/min. Trios software was used to perform the data analysis. Gel fraction measurements were carried out to monitor the degree of cross-linking by placing samples in hot xylene at 120 °C for 24 h and comparing the initial and final mass to determine the insoluble fraction. Fourier transform infrared (FTIR) spectroscopy was carried out with a Thermo Scientific Nicolet iS50 instrument with a resolution of 4 cm^–1^, a scan range of 4000–600 cm^–1^, and an average of 32 scans. Dimensional change was assessed by comparing the final carbon’s critical dimensions to those of the initial printed structure, the error bars of which were determined by three replicates. Carbon yield was determined by comparing the initial printed structure’s mass to the final carbon (following subtraction of the mass corresponding to the carbon black (∼5 wt %)). The apparent density of the carbonized structures was calculated by dividing the mass of the structure, determined by analytical balance, by the volume. Thermogravimetric analysis (TGA) was conducted with a Discovery 550 instrument (TA Instruments), where carbon samples were heated under a nitrogen environment, while carbon–cobalt composites were heated under air, both with a rate of 10 °C/min.

To characterize the pore textures of the structured carbons, nitrogen physisorption measurements were carried out with a Tristar II 3020 instrument (Micromeritics) at 77 K. Pore size distributions were obtained with nonlocal density functional theory for carbon slit pores, and surface areas were determined using Brunauer–Emmett–Teller analysis. X-ray diffraction (XRD) was performed with a Rigaku MiniFlex XRD with Cu Kα radiation with a 2θ range of 15°–70° and a scan speed of 1°/min. Electrical conductivity was measured using the two-point method with a Keithley 2400 source meter. Electrical contact was made by using copper wires with rectangle-shaped samples. Electrochemical measurements were carried out in a three-electrode system, with the 3D-printed structured carbon or carbon–cobalt composite as the working electrode, Ag/AgCl as te reference electrode, and platinum wire as the counter electrode, and all tests were completed in a 1 M KOH aqueous electrolyte. Cyclic voltammetry (CV) and galvanostatic charge–discharge (GCD) measurements were carried out with a CHI 660D electrochemical workstation. CV was measured at different scan rates ranging from 5 to 100 mV/s. GCD was measured at different current densities from 0.2 to 1.0 A/g. Specific capacitance was calculated based on both CV and GCD tests, using the following two equations (eq 1 and eq 2), respectively:

$$C_{s}=\frac{\int_{V_{1}}^{V_{2}}I\;dV}{m\nu \left(V_{2}-V_{1}\right)}$$ 1

where *C*_s_ is the gravimetric capacitance, *V*_1_ and *V*_2_ are the lowest and highest limit of the potential window, respectively, *m* is the total weight of the electrode, and ν is the scan rate in the CV test.

2 $$C_{s}=\frac{I\Delta t}{m\Delta V}$$

where *I* is the constant current during the test, Δ*t* is the total discharge time, *m* is the total weight of the electrode, and Δ*V* is the potential drop over the discharging process.

## 3 Results and Discussion

The PE microparticles for SLS 3D-printing were prepared by a temperature-induced phase separation (TIPS) method (Figure 1a,b). The liquid–liquid phase separation followed by crystallization of PE during the cooling process results in solid PE microparticles. PE microparticles were then mixed with a small fraction (5 wt %) of carbon black (CB) nanoparticles. The introduction of CB enhances the optical absorption of microparticles during the SLS printing process, though it can also enhance the electrical conductivity of the final carbon structures as will be discussed later.

**Figure 1:**
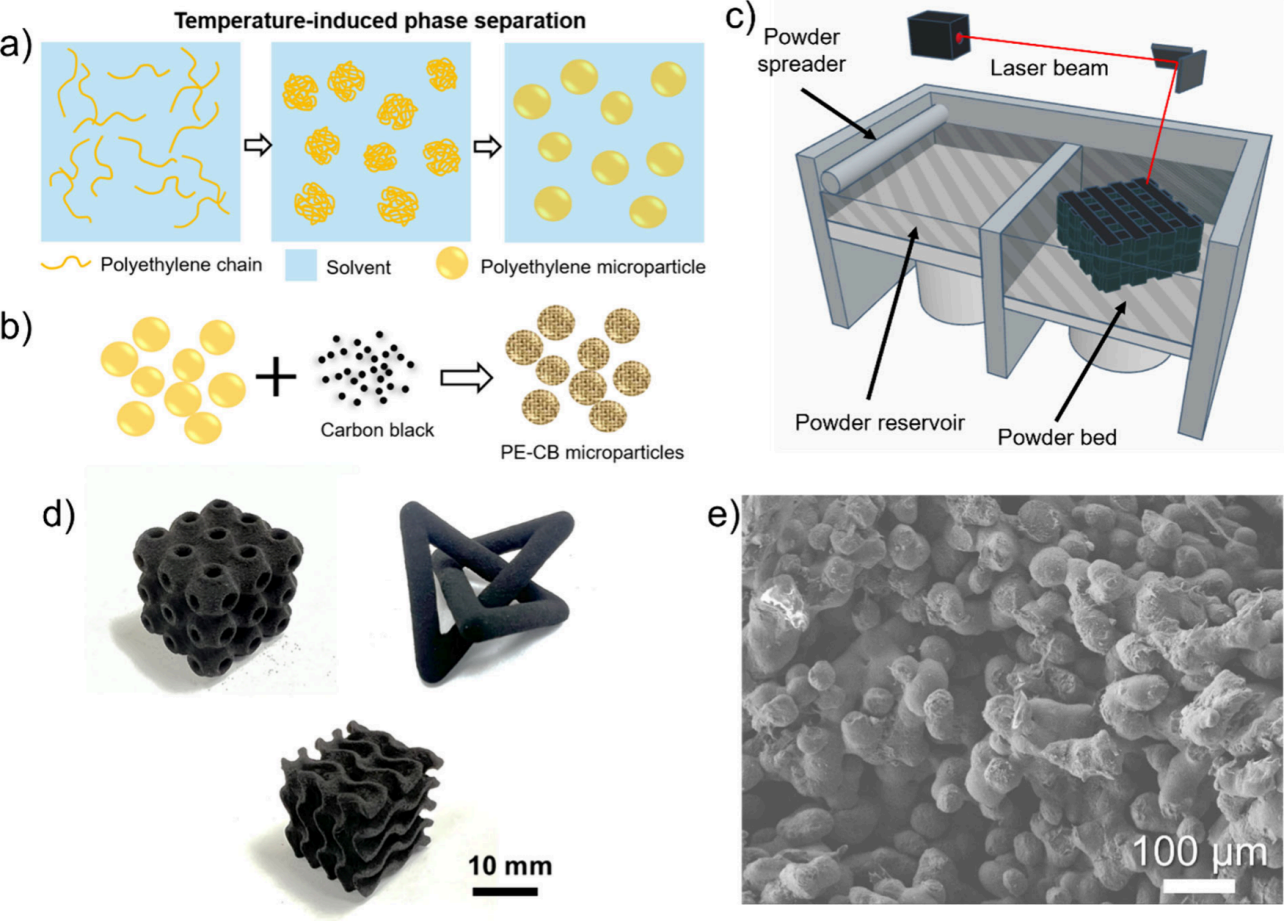
(a) Scheme of the preparation of PE microparticles by the TIPS method. (b) Scheme of the preparation of PE-CB microparticles after mixing PE with CB. (c) Schematic representation of the SLS 3D-printing process. (d) Photos of the representative SLS-printed structures from PE microparticles (containing 5% CB). (e) Cross-sectional SEM image of the SLS-printed structures from PE (containing 5% CB).

The SLS process involves laser-induced sintering of preheated PE (with CB) microparticles in selective locations (Figure 1c). After one layer is completed, a fresh microparticle layer is deposited and spread by a roller, which is followed by sequential SLS. Such a process is repeated until the entire 3D structure manufacturing is completed. To demonstrate the structural design space achievable through this printing strategy, Figure 1d shows several printed structures with relatively complex geometries, including a lattice cube, gyroid, and trefoil knot. These are PE composites containing CB. The internal microstructure of printed PE samples can be examined through SEM imaging as shown in Figure 1e, where microparticles are observed to partially coalesce, forming a continuous macrostructure. As previously reported,^40^ the porosity of printed structures can be tailored during the SLS printing process. Specifically, incomplete sintering of PE microparticles introduces inherent porosity within printed structures, which can be modulated through printing parameters and carbon black loading. This continuous, hierarchically porous morphology can facilitate their use as supporting/host materials for molecular transport and sorption, enabling a broad range of applications such as sorbents for CO_2_ capture,^52,53^ catalysis,^54,55^ and biomedical applications.^56^

Figure 2a illustrates the process of converting SLS-printed PE samples into structured carbon. In the sulfonation-induced cross-linking reaction, sulfonic acid groups are introduced onto the polymer backbone of PE, which then undergo homolytic dissociation, forming olefins that participate in a series of addition and rearrangement reactions. This reaction mechanism has been well investigated and reported in the literature,^57–59^ which ultimately leads to intermolecular cross-linking throughout the printed structure. Subsequently, pyrolysis under an inert atmosphere transforms the cross-linked polymers into structured carbon materials. The PE sulfonation progress can be tracked with DSC measurements through monitoring the loss in the degree of crystallinity, as introducing bulky sulfonic acid inhibits polymer recrystallization (Figure S1 and Figure 2b). A steady decrease in the degree of crystallinity was observed until a nearly amorphous morphology was reached following 18 h of reaction time. Gel fraction measurements were conducted, as shown in Figure 2c, to further examine the degree of cross-linking of polymer samples as a function of time, showing an insoluble content of 22, 52, and 81 wt % following 2, 8, and 18 h, respectively. It is worth noting that the neat material exhibits an insoluble fraction of 17%, which can be attributed to the loading of carbon black (5 wt %) and slight laser-induced cross-linking of PE during SLS printing.^60^ A plateau value of 89 wt % was reached following 24 h, while macrostructures were retained following extraction of the solvent, indicating sufficient cross-linking of the bulk structure. Fourier transform infrared (FTIR) spectroscopy was conducted to assess the shifts in chemical composition during the cross-linking reaction, as shown in Figure S2. Upon sulfonation for 2 h, a broad band associated with hydroxyl stretching appears at ∼3350 cm^–1^, in addition to bands corresponding to sulfonic acid groups between 1060 to 1000 cm^–1^. As the reaction progresses, the band corresponding to alkyl stretching diminishes significantly following 2 h and is nearly absent at all longer reaction times. Moreover, alkene stretching (1600 cm^–1^) appears following 2 h and becomes more pronounced with longer reaction times. After 8 h of reaction, the FTIR spectra exhibit minimal shifts following longer reaction times.

**Figure 2:**
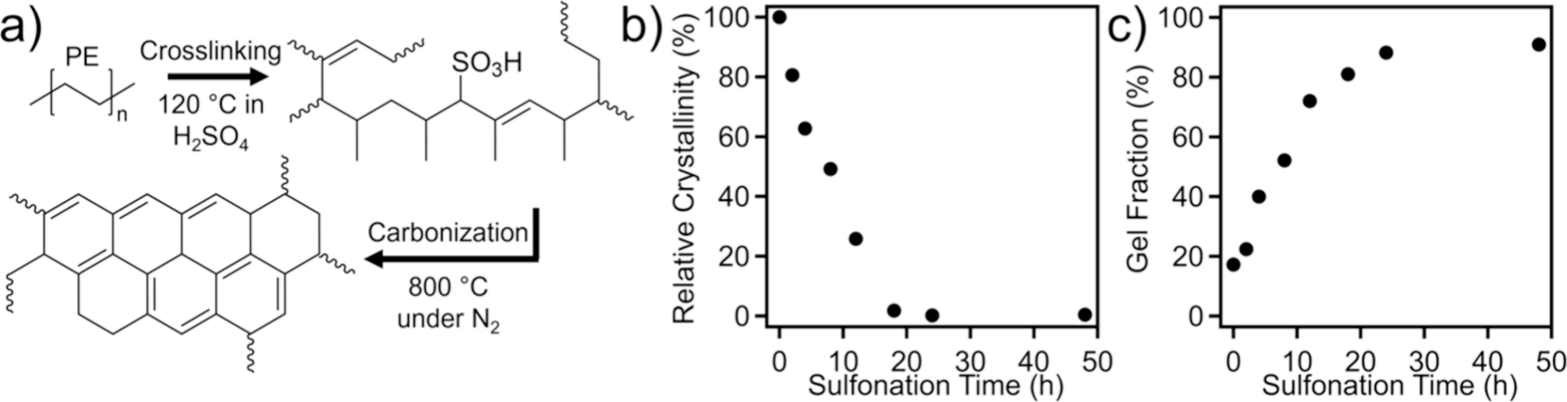
(a) Simplified reaction mechanism for the conversion of SLS-printed PE to carbon products through sulfonation-induced cross-linking and subsequent pyrolysis. (b) Relative degree of crystallinity and (c) gel fraction of SLS-printed PE structures as a function of sulfonation time at 120 °C.

The internal microstructure of SLS-printed PE samples following various reaction times was probed through SEM imaging, as shown in Figure 3. It was observed that the structure undergoes densification with increased reaction time, as has been reported with PP-derived carbon fibers.^61^ While the densification of the printed structure increases with increased reaction time, hierarchical porosity is still retained at long reaction times, indicating that porosity can be further tuned through modulating reaction conditions. Previous polyolefin-derived structured carbons prepared from FFF printing have exhibited micron-sized crack formation following sulfonation-induced cross-linking.^35^ This is because sulfonation of PE is diffusion-controlled, causing the outer layers of printed structures to become hydrophilic and swell in sulfuric acid, while the inner bulk remains hydrophobic. This differential swelling generates stress, leading to crack formation. In SLS-printed PE structures, it was found that no microscopic crack formation occurred, which could be due to the smaller path length of SLS-printed microparticles (∼35 ± 9 μm) compared to FFF-printed layer resolutions (∼0.4 mm), in addition to the high porosity observed in the SLS-printed system, further promoting sulfuric acid diffusion. We note that the absence of crack formation during sulfonation-induced cross-linking has been observed in structured polyolefins with smaller critical dimensions,^62–64^ such as within PP textiles and PE fibers with a diameter of less than 50 μm.

**Figure 3:**
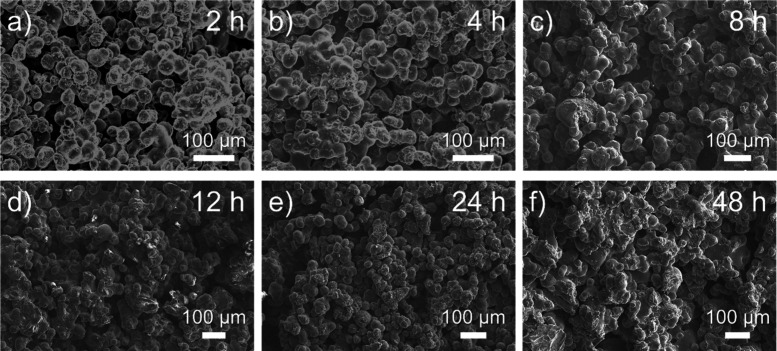
SEM micrographs of SLS-printed PE cross sections following sulfonation at 120 °C for (a) 2, (b) 4, (c) 8, (d) 12, (e) 24, and (f) 48 h.

Figure 4a shows that increasing cross-linking reaction time resulted in a steady increase in carbon yield reaching up to 62 wt % (compared to the printed structure’s initial mass) following 24 h. In comparison to FFF-derived carbon structures,^35^ the SLS-printed PE demonstrates comparable yields with lower reaction temperatures, which may be due to sulfuric acid diffusion being facilitated by the smaller diffusion path length and hierarchical porosity of the SLS-printed structure. Moreover, Figure S3 shows thermogravimetric analysis (TGA) results for the neat material with a carbon yield of 6.6 wt %, correlating closely with the initial carbon black loading content. The dimensional change of structured carbons (compared to initial dimensions) was then assessed with several initial printed dimensions to determine the scalability of this process (from 0.5 to 1.75 cm), shown in Figure 4b. It was found that the in-plane dimensional shrinkage (*X*- and *Y*-directions along printing deposition) was approximately 11%, while the out-of-plane shrinkage (*Z*-direction, normal to printing deposition) was approximately 8%. These results are consistent with varied initial sample size, indicating the scalability of this process with control over the macroscopic structures of resulting materials. We note that SLS-derived structured carbons exhibit isotropic shrinkage, which is distinct from their counterparts prepared by the FFF process, which show approximately 20% in-plane and 5–9% out-of-plane shrinkage. The anisotropic dimensional shrinkage in FFF structures arises from layer-by-layer addition along the *Z*-axis. In contrast, our SLS-printed structures maintain isotropic shrinkage during polymer-to-carbon conversion, which is similar to other systems that employ VP of carbon precursors followed by pyrolysis.^30^ Moreover, the apparent density of the final carbon structure was found to be 0.37 g/cm^3^, demonstrating the structured carbon’s highly porous framework. Figure 4c displays the versatility of SLS-printed structured carbons with varied structural complexities, including a lattice cube, trefoil knot, and gyroid structure. These are the printed PE samples (Figure 1d) after cross-linking and pyrolysis where they become fully carbon structures. Through this SLS approach, increased printing resolution can be achieved, which allows for the fabrication of complex structures with isotropic shrinkage and smaller feature sizes.

**Figure 4:**
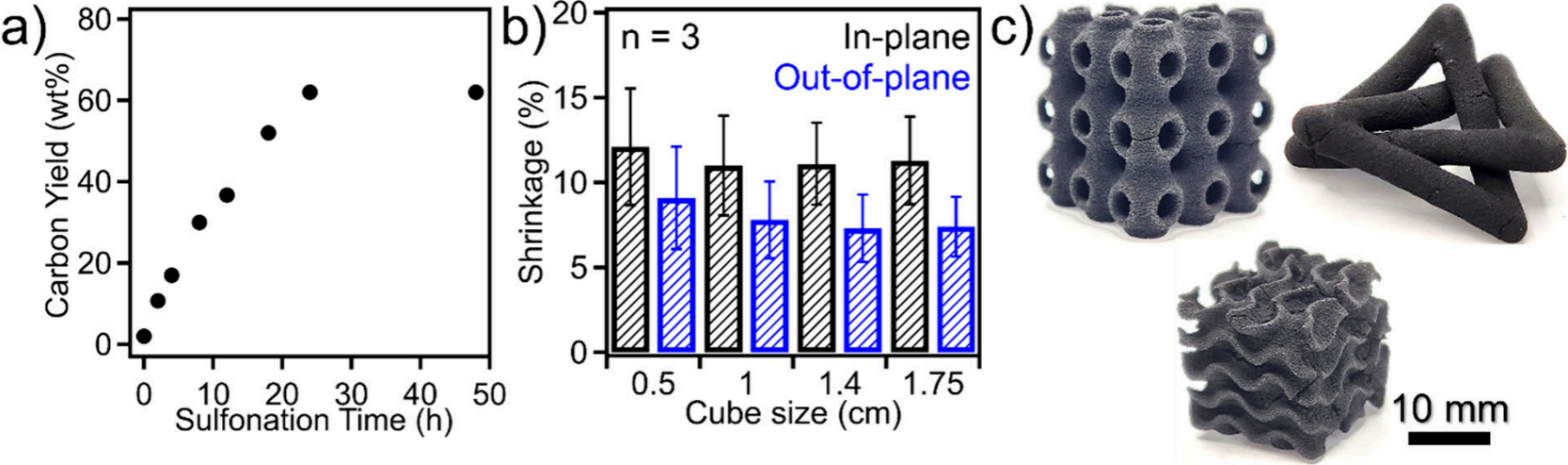
(a) Carbon yield as a function of sulfonation time for SLS-printed structures, (b) dimensional shrinkage of carbonized structures as a function of printed size, and (c) demonstration of fully carbon complex structures attainable through the reported method.

Following pyrolysis, the morphology of the SLS-derived structured carbon was studied, as shown in Figure 5. At low reaction times (2 and 4 h), hollow carbon microparticles can be seen with noticeable distortion compared to their initial spherical structure. Following 8 h of reaction, the hollow character is no longer observed on the surface, and longer reaction times (12, 24, and 48 h) showcase increased densification among distinct microparticles found in the cross-linked counterparts. These results confirm that the cross-linking reaction was from the outside inward. Furthermore, micron-sized cracks are not present following pyrolysis at all reaction times. However, as seen in the images in Figure 4c, macrocracks can be observed, primarily associated with handling structures following pyrolysis. We note that fabrication of hollow microspheres through this strategy at low sulfonation times, though with reduced carbon yields and structural retention, may be a promising route to fabricating carbon spheres with complex architectures and improved functionalities. A previous report shows the synthesis of carbon microspheres has been previously achieved through the formation of poly­(styrene-*co*-butyl acrylate)/polypyrrole (PSBA/PPy) latex particles followed by pyrolysis,^65^ exhibiting a high surface area of ∼500 m^2^/g. Our method might provide a promising route for preparing hollow carbon microspheres coupled with a 3D complex macrostructure.

**Figure 5:**
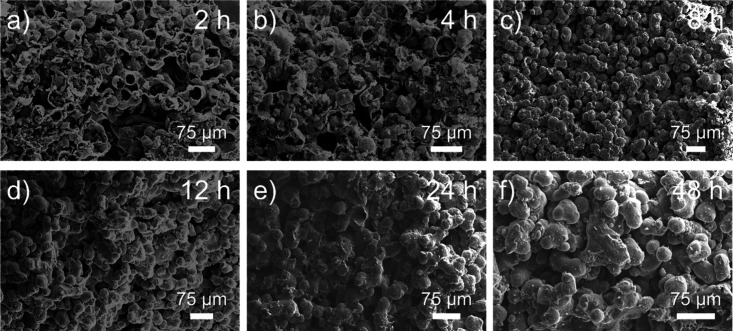
SEM micrographs of carbonized PE cross sections following sulfonation at 120 °C for (a) 2, (b) 4, (c) 8, (d) 12, (e) 24, and (f) 48 h.

Nitrogen physisorption measurements were conducted to assess how the pore characteristics of SLS-derived structured carbon change as a function of cross-linking time. Specifically, Figure 6a presents the surface area as a function of sulfonation time, where the carbon derived from the sample that was cross-linked for 2 h has a surface area of 303 m^2^/g, which continues to gradually increase and plateau at ∼690 m^2^/g following 48 h of reaction time. Improved surface area from increasing cross-linking degree of polyolefin precursors was also found in a previous report.^66^ Furthermore, the pore volume across all reaction times ranges between 0.45 and 0.65 cm^3^/g. Figure S4a,b shows the nitrogen sorption isotherms and pore size distributions for carbons prepared from various sulfonation reaction times. Carbons prepared with low degrees of cross-linking were observed to have a broad macro/mesoporous population, which gradually transitions to a more microporous-dominated porous carbon texture at increased cross-linking time. The nitrogen adsorption–desorption isotherm for the carbon (which was cross-linked for 48 h) is depicted in Figure 6b, where a type II isotherm was observed, indicating the presence of both micro- and macropores. Figure 6c presents the pore size distribution of the sample derived from the NLDFT model, revealing a predominant presence of micropores, along with a minor fraction of mesopores averaging 4.7 nm, and a shoulder at larger pore sizes.

**Figure 6:**
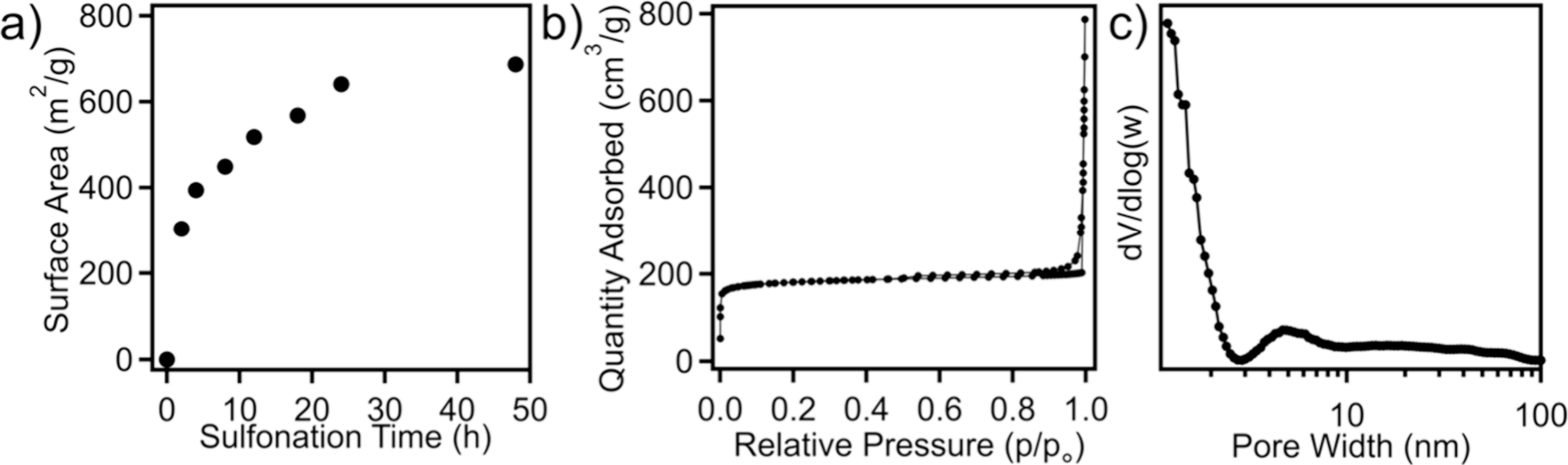
(a) Surface area of SLS-printed carbon as a function of sulfonation time. (b) Nitrogen physisorption isotherm and (c) corresponding pore size distribution for an SLS-printed carbon (with a 48 h cross-linking reaction time).

To further extend the functionality of SLS-derived structured carbon, a simple metal infiltration step was introduced during the fabrication process, as illustrated in Figure 7a. Specifically, cross-linked structures were immersed in a cobalt nitrate solution, where sulfonic acid groups along the polymer chain interact with and bind metal ion species.^66^ Upon pyrolysis, metal nitrates are converted to metal oxide particles to form carbon–metal composites, allowing for a wide range of material properties which can enable an expanded application space.^67–71^ In this work, cobalt oxide was chosen as a model system due to its relatively high theoretical specific capacitance (∼3560 F/g).^72^ To improve the conductivity of carbon–cobalt nanocomposites, pyrolysis was performed under nitrogen up to 1300 °C with a 3 h time hold. Through modulating the cobalt nitrate solution concentration from 0.5 to 5 M, cobalt loading levels in the final carbon matrix can be increased from 7, 11, 20, to 31 wt % with 0.5, 1, 2, and 5 M, respectively (Figure S5 and Figure 7b). Cobalt loading levels were determined from TGA measurements in air, where carbon can be completely decomposed. Furthermore, to understand the extent of cobalt functionalization on the structured carbon, energy-dispersive X-ray spectroscopy (EDS) was conducted. Figure 7d and Figure S6 show cobalt in red, where the carbon surface in general is broadly covered in cobalt.

**Figure 7:**
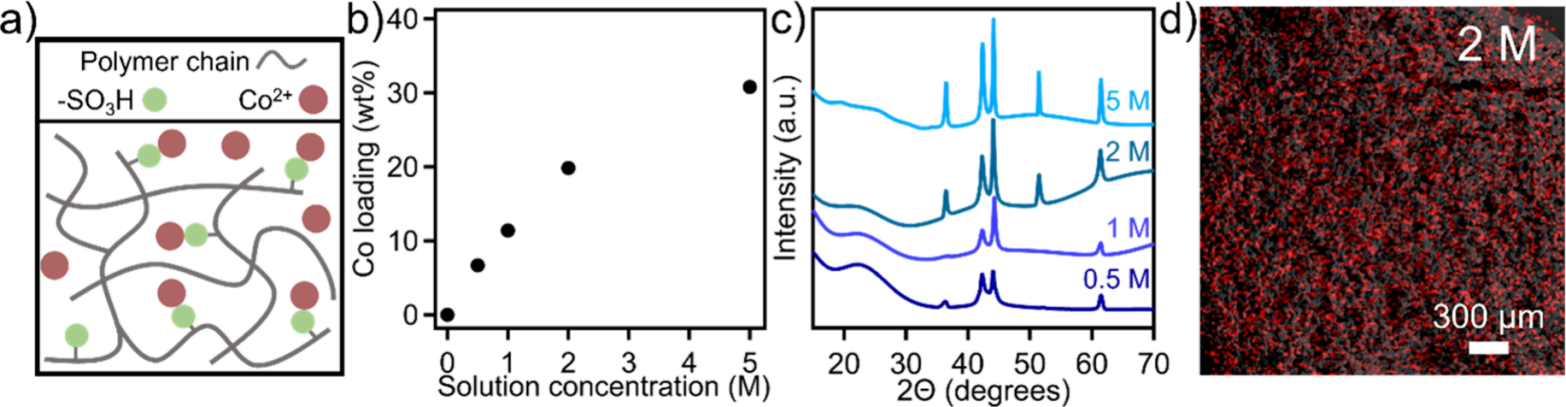
(a) Schematic illustration of the carbon–metal nanocomposite fabrication process. A sulfonated structure is submerged in a metal nitrate solution, allowing for infusion of the target metal, and subsequent pyrolysis forms the final carbon–metal nanocomposite. (b) Cobalt loading as a function of solution concentration. (c) Representative XRD patterns for carbon–cobalt nanocomposites as a function of metal nitrate solution concentration. (d) EDS cobalt elemental map for a carbon–cobalt nanocomposite prepared from a 2 M cobalt nitrate solution (cobalt in red).

Pore characteristics of these composites were studied through nitrogen physisorption, as shown in Figure S7, where the surface area decreases from 687 m^2^/g in the neat carbon to 532, 527, and 330 m^2^/g with 11, 20, and 31 wt % cobalt loading, respectively. Similarly, the pore volume decreases from 0.64 cm^3^/g for carbon to approximately 0.40 cm^3^/g, which remains consistent across all cobalt loadings in the nanocomposites. Representative X-ray diffraction (XRD) patterns are presented in Figure 7c for the nanocomposites with various cobalt loadings, where a broad peak at ∼23.5° is observed for all samples corresponding to amorphous carbon with an interlayer distance of 3.8 Å. Additionally, peaks at 36.4°, 42.3°, and 61.4° correspond to the (111), (200), and (220) reflections of cobalt­(II) oxide (CoO), while a peak at 44.1° may correspond to the (400) reflection of cobalt­(II,III) oxide (Co_3_O_4_).^73,74^ As cobalt loading increases, all peak intensities increase, while an additional peak at 51.4° appears corresponding to the (211) reflection of cobalt­(IV) oxide for the nanocomposites with the highest cobalt loadings (20 and 31 wt %).^75^ The formation of cobalt dioxide at higher cobalt loadings may be attributed to saturation of available surface sites, resulting in greater interactions between cobalt species and the formation of cobalt dioxide phases. It should also be noted that the baseline begins to deviate at increased cobalt loadings, which may be associated with increased noise from cobalt fluorescence with a copper radiation source. We also studied the electrical conductivity of the structured carbon and carbon–cobalt composites. The results (Figure S8) showed that the structured carbon has an electrical conductivity of 240 S/m, and the structured carbon–cobalt composite has an electrical conductivity of 700 S/m. The enhanced conductivity of the carbon–cobalt nanocomposite is mainly due to its reduced porosity and pore volume.

The electrochemical performance of the SLS-derived structured carbon and carbon–cobalt composites was systematically evaluated using cyclic voltammetry (CV) and galvanostatic charge–discharge (GCD). Electrochemical testing was conducted in a three-electrode setup with the 3D-structured carbon or carbon–cobalt composite (containing 20 wt % cobalt particles) as the working electrode. The reference electrode was Ag/AgCl, the counter electrode was a platinum wire, and a 1 M KOH was used as the liquid electrolyte. Figure 8a shows CV scan results (at a rate of 5 mV/s) of the structured carbon and carbon–cobalt nanocomposite. The structured carbon electrode shows a smooth curve over the entire scan range without obvious peaks. The CV curve shape is a quasi-rectangle in the range of −0.2 to 0.2 V, which indicates typical electrochemical double-layer capacitive (EDLC) behavior. The structured carbon–cobalt nanocomposite exhibits prominent redox peaks at approximately 0.2 and −0.6 V vs Ag/AgCl. These peaks are an indication of faradaic reactions, most likely from the reversible Co^2+^/Co^3+^ and Co^3+^/Co^4+^ redox transitions, which provide pseudocapacitive contribution in addition to the electric double-layer capacitance.^76–78^ At relatively low scan rates, the specific capacitance calculated from the enclosed area under the CV curve of the carbon–cobalt composite is significantly higher than that of carbon. For instance, at a scan rate of 5 mV/s, the carbon–cobalt composite has a 92 F/g capacitance, which is about 1.5 times higher than that of the carbon (61 F/g). The differences will be discussed further in the next paragraph. We also compared the capacitance with literature reports on similar material systems (Table S1) and showed that our system has relatively good performance.

**Figure 8:**
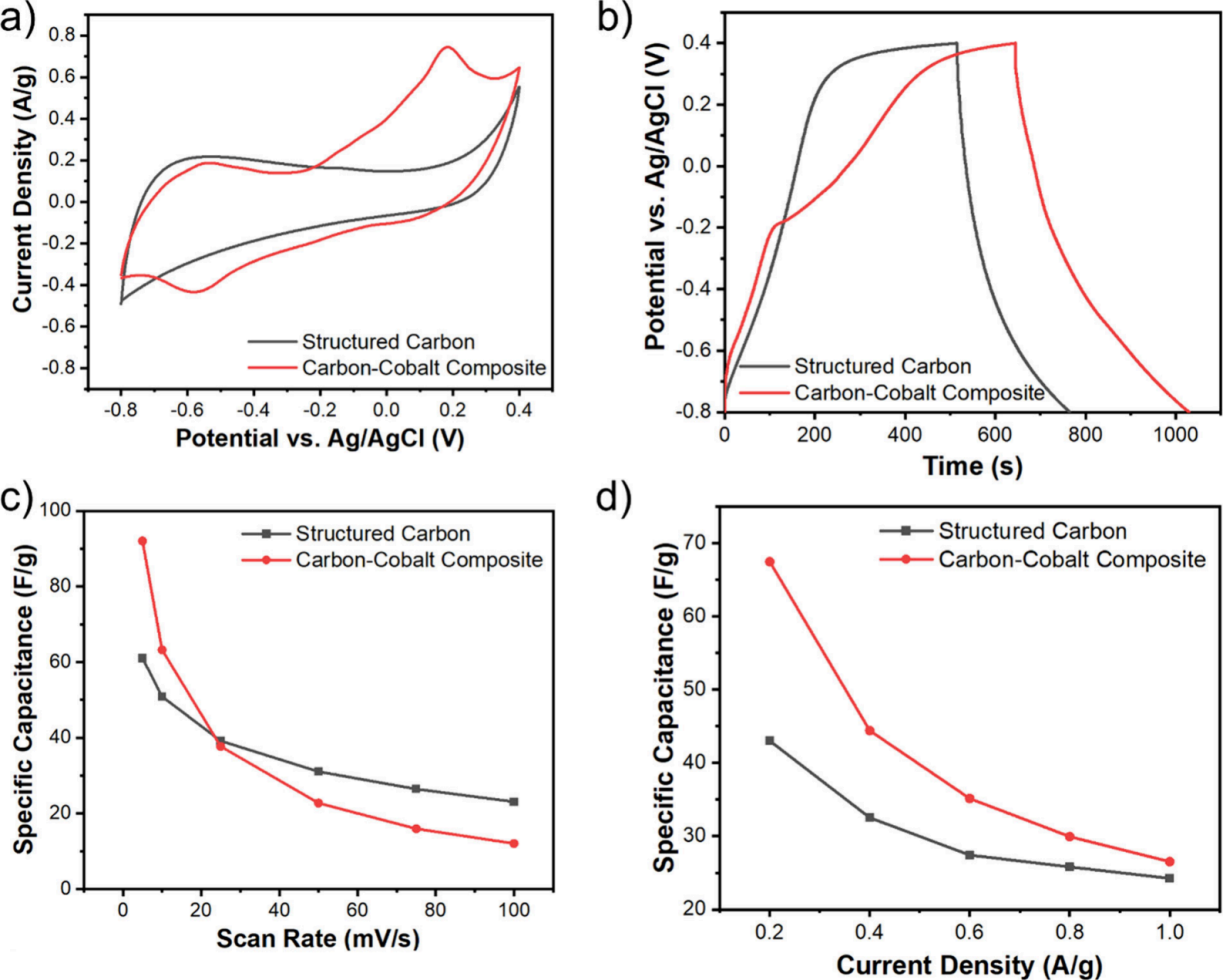
Electrochemical characterization of the structured carbon and the carbon–cobalt composite. (a) CV scans of the structured carbon and carbon–cobalt composite at a scan rate range of 5 mV/s. (b) GCD curves of the structured carbon and carbon–cobalt composite at a current density of 0.2 A/g. (c) The specific capacitance for the structured carbon and carbon–cobalt composite at different scan rates calculated from CV results. (d) The specific capacitance for the structured carbon and carbon–cobalt composite at different current densities calculated from GCD results.

The GCD curves for the structured carbon and carbon–cobalt composites (at a current density of 0.2 A/g) are shown in Figure 8b. It can be observed that the structured carbon shows quasi-triangular-shaped charge–discharge behavior. A relatively fast charging to 0.24 V is followed by a slower charging to 0.40 V, with a total discharge time of about 150 s. In comparison, the structured carbon–cobalt composite shows a turning point at about 0.2 V during the charging process, which is characteristic of pseudocapacitive behavior and matches with the oxidation peak in CV measurements. The total discharge time is about 380 s, which is substantially larger than that of the structured carbon. Moreover, the specific capacitance from GCD testing, measured at a currently density of 0.2 A/g, was higher for the carbon–cobalt composite (67 F/g) than carbon (43 F/g). Furthermore, the electrochemical performance of the structured carbon was found to be less dependent on the scan rate or current density than that of the carbon–cobalt composite. While there are minimal shifts in the carbon’s CV scans across various scan rates (Figure S9a), the redox peaks for the carbon–cobalt composite become less obvious at higher scan rates (Figure S9b).

Additionally, the specific capacitance of the carbon–cobalt composite has a larger drop with increased scan rate or current density than that of the carbon (Figure 8c,d and Figure S9c,d). We hypothesize that the primary cause of this phenomenon is two-fold. First, the carbon–cobalt composite exhibits reduced porosity and pore volume (0.40 vs 0.64 cm^3^/g) compared to the structured carbon, which can restrict ion diffusion and hinder redox reaction kinetics at high scan rates. Second, the structured carbon electrode primarily relies on EDLC, whereas the carbon–cobalt composite benefits from both EDLC and faradaic capacitance. EDLC typically shows minimal dependence on scan rate due to its electrostatic charge storage mechanism, whereas faradaic capacitance is significantly affected by scan rate, as it involves reversible redox reactions occurring at the electrode surface (Figure S10).^79^ Nevertheless, these electrochemical measurement results strongly demonstrate the ability of our method to directly fabricate 3D architected, carbon-based electrodes for energy storage applications using low-cost precursors and scalable processes. Our approach offers significant advantages by enabling control over pore texture, composition, and macroscopic morphology, paving the way for the development of cost-effective energy storage solutions.

## 4 Conclusions

This work demonstrates the fabrication of carbons and their composites with hierarchical porosity by employing SLS-printed PE as the starting material. The impact of sulfonation-induced cross-linking on the resulting carbon structures was systematically studied, including both particle coalescence and reaction kinetics. Pyrolysis of PE structures with low cross-linking resulted in hollow microsphere carbon structures due to a diffusion-controlled sulfonation process, while fully cross-linked samples yielded structured carbons with up to 62 wt % yield and nearly isotropic dimensional shrinkage (∼8–11%). Additionally, carbon–metal nanocomposites were fabricated by embedding metal nitrates in cross-linked samples via a simple immersion process followed by pyrolysis. Electrochemical evaluation of the structured carbon and carbon–cobalt nanocomposites as electrode materials showed that the carbon–cobalt composites exhibited superior performance at low scan rates due to their combined EDLC and pseudocapacitive properties. However, at high scan rates, their lower porosity and redox kinetics led to a larger capacitance decline. The ability to precisely control the 3D geometries, porosity, and chemical compositions of these 3D-printed carbon and carbon–metal composites presents promising opportunities for advanced energy storage applications.

## Supplementary Material



## Data Availability

The data supporting this study are available upon request from the corresponding authors.

## Notes

DSC thermograms, FTIR spectra, and TGA thermograms for neat SLS-printed PE and samples cross-linked for various amounts of time; nitrogen sorption isotherms and pore size distributions for structured carbons prepared with various cross-linking times; TGA thermograms, EDS cobalt elemental maps, nitrogen sorption isotherms, and pore size distributions for various carbon–cobalt composites; *I*–*V* curves, CV scans, GCD curves, capacitance retention as a function of scan rate, and capacitance retention as a function of current density; and comparison of electrochemical performance of materials in this study and various similar material systems from the literature (PDF)
