# Supplementary material for: On-Demand Porous Carbon Fabrication via Selective Laser Sintering for Electrochemical Energy Storage
Source: ACS Appl Eng Mater. 2025 Aug 7;3(8):2391–401. doi: 10.1021/acsaenm.5c00297 (PMC12379160; doi:10.1021/acsaenm.5c00297)
Supplement: Supplementary file 1 [file em5c00297_si_001.pdf]

Supporting Information:

On-Demand Porous Carbon Fabrication via Selective Laser Sintering for Electrochemical Energy Storage

Anthony Griffin,[a,†] Muxuan Yang,[b,†] Parker Frame,[a] Weinan Xu,[b,*] Zhe Qiang[a,*]

[a]School of Polymer Science and Engineering, University of Southern Mississippi, Hattiesburg, MS, 39406, USA

[b]School of Polymer Science and Polymer Engineering, The University of Akron, Akron, OH 44325, USA

† Indicates equal contributions from both authors

Corresponding authors: Z. Q. (zhe.qiang@usm.edu), W.X. (weinanxu@uakron.edu)

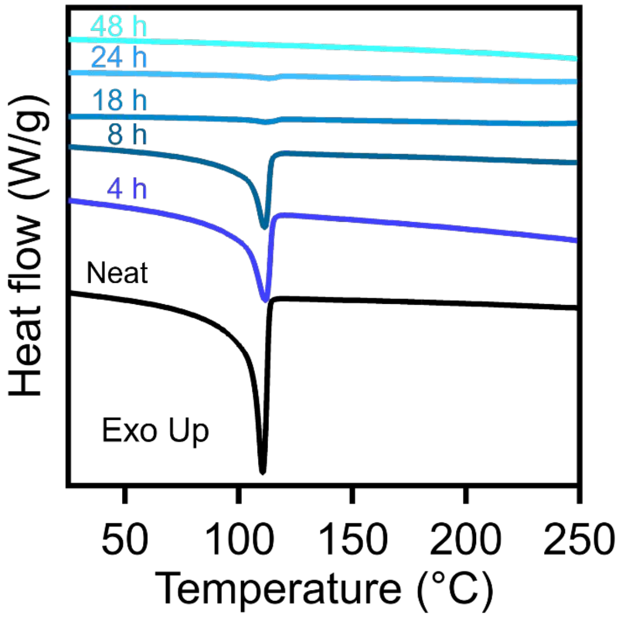

Figure S1. DSC thermograms of the second heating trace for SLS-printed PE sulfonated at 120 °C as a function of reaction time.

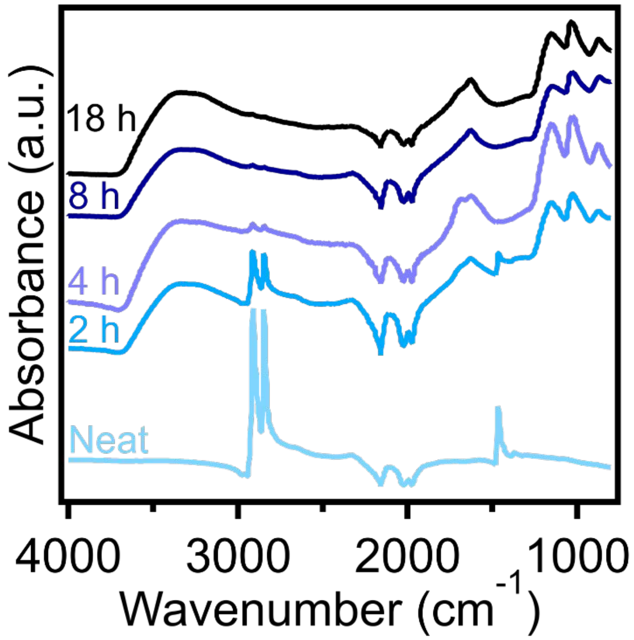

Figure S2. FTIR spectra for SLS-printed PE sulfonated at 120 °C as a function of reaction time.

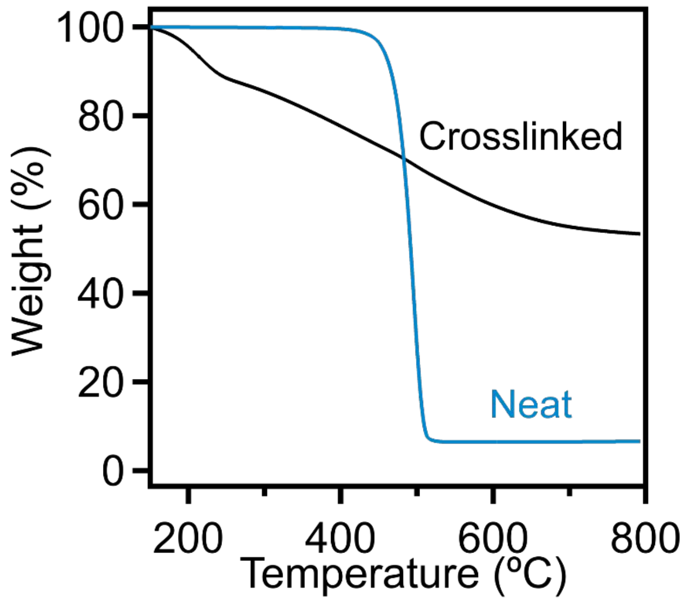

Figure S3. TGA thermograms up to 800 °C in N_2 atmosphere for PE following printing and following crosslinking for 48 h.

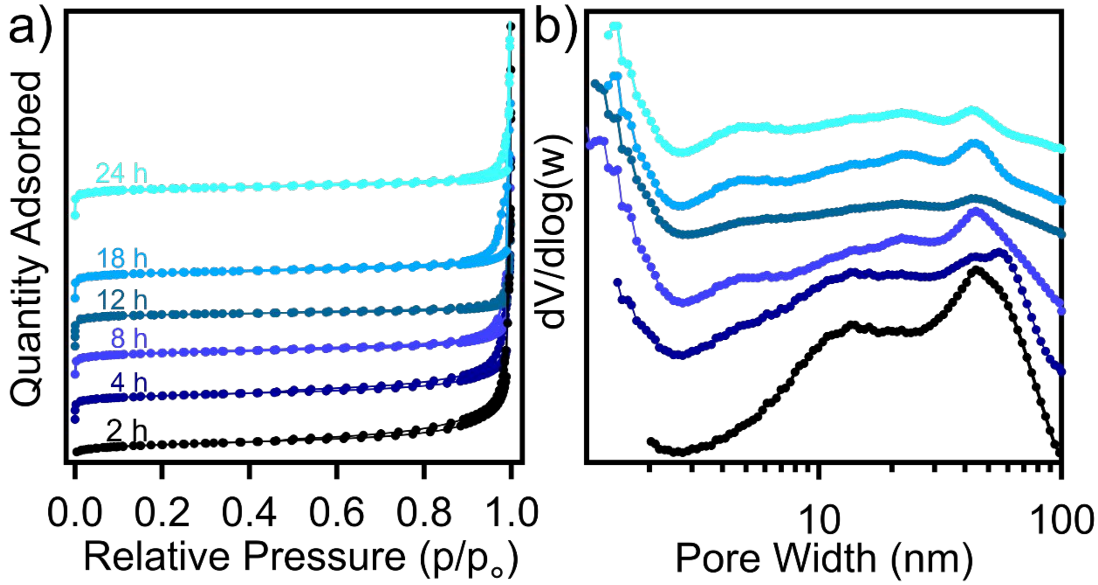

Figure S4. a) Nitrogen sorption isotherms and b) pore size distributions for SLS-derived carbon prepared from various crosslinking times.

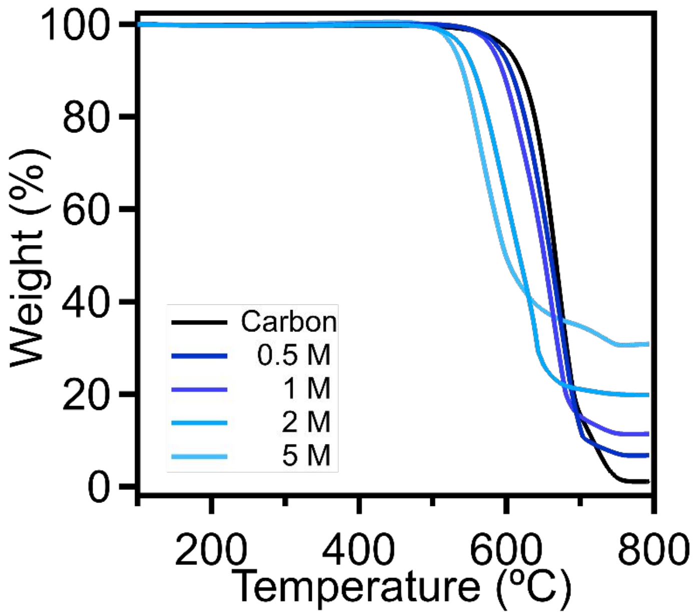

Figure S5. TGA thermograms up to 800 °C under air for carbon-cobalt nanocomposites prepared from various metal nitrate solution concentrations.

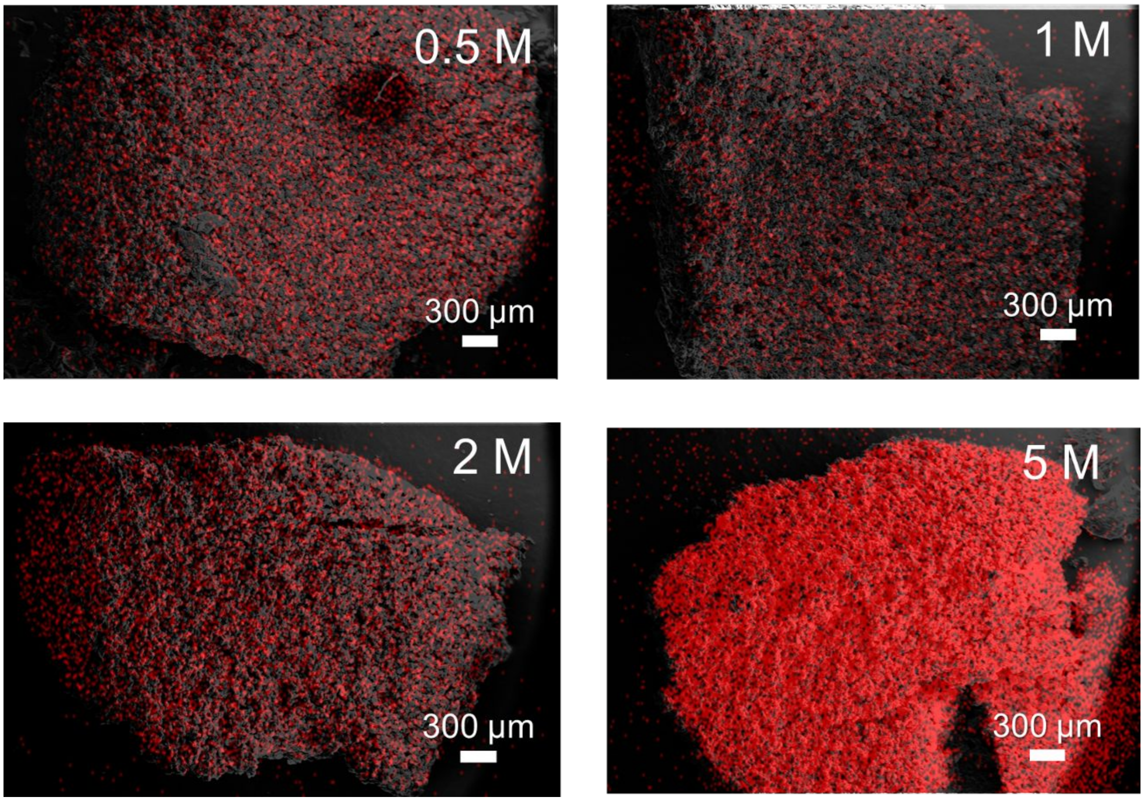

Figure S6. EDS cobalt elemental maps for carbon-cobalt nanocomposites prepared from various metal nitrate solution concentrations (cobalt in red).

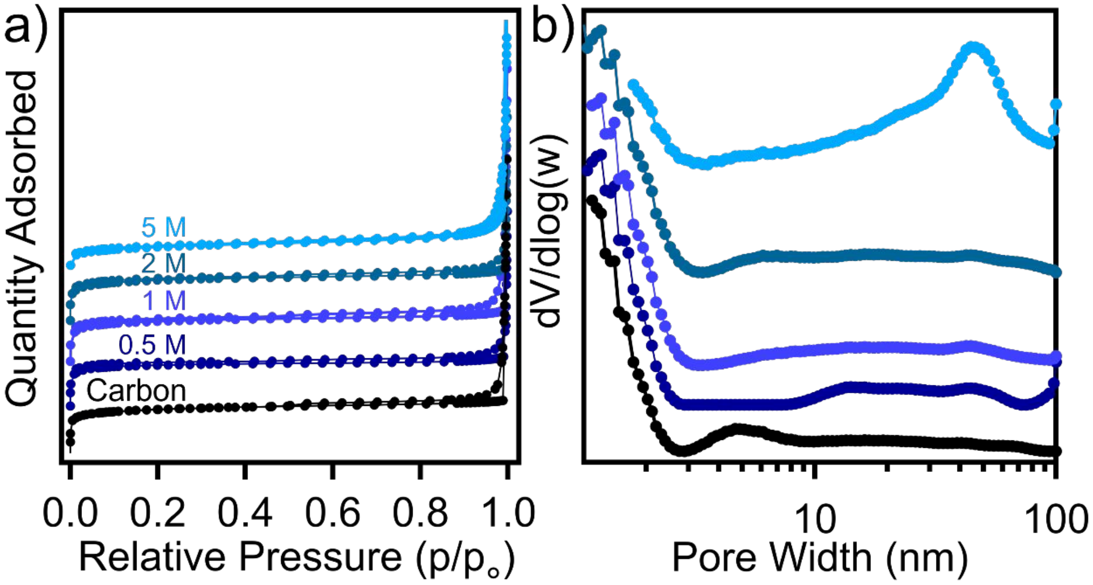

Figure S7. a) Nitrogen sorption isotherms and b) pore size distributions for SLS-derived carbon-cobalt nanocomposites prepared from various metal nitrate solution concentrations.

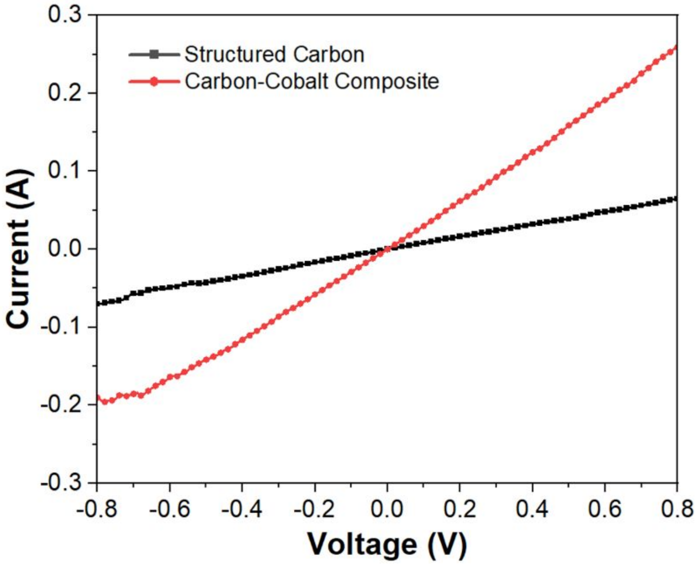

Figure S8. I-V curves of the structured carbon and carbon-cobalt composite fabricated by SLS 3D printing followed by crosslinking and carbonization.

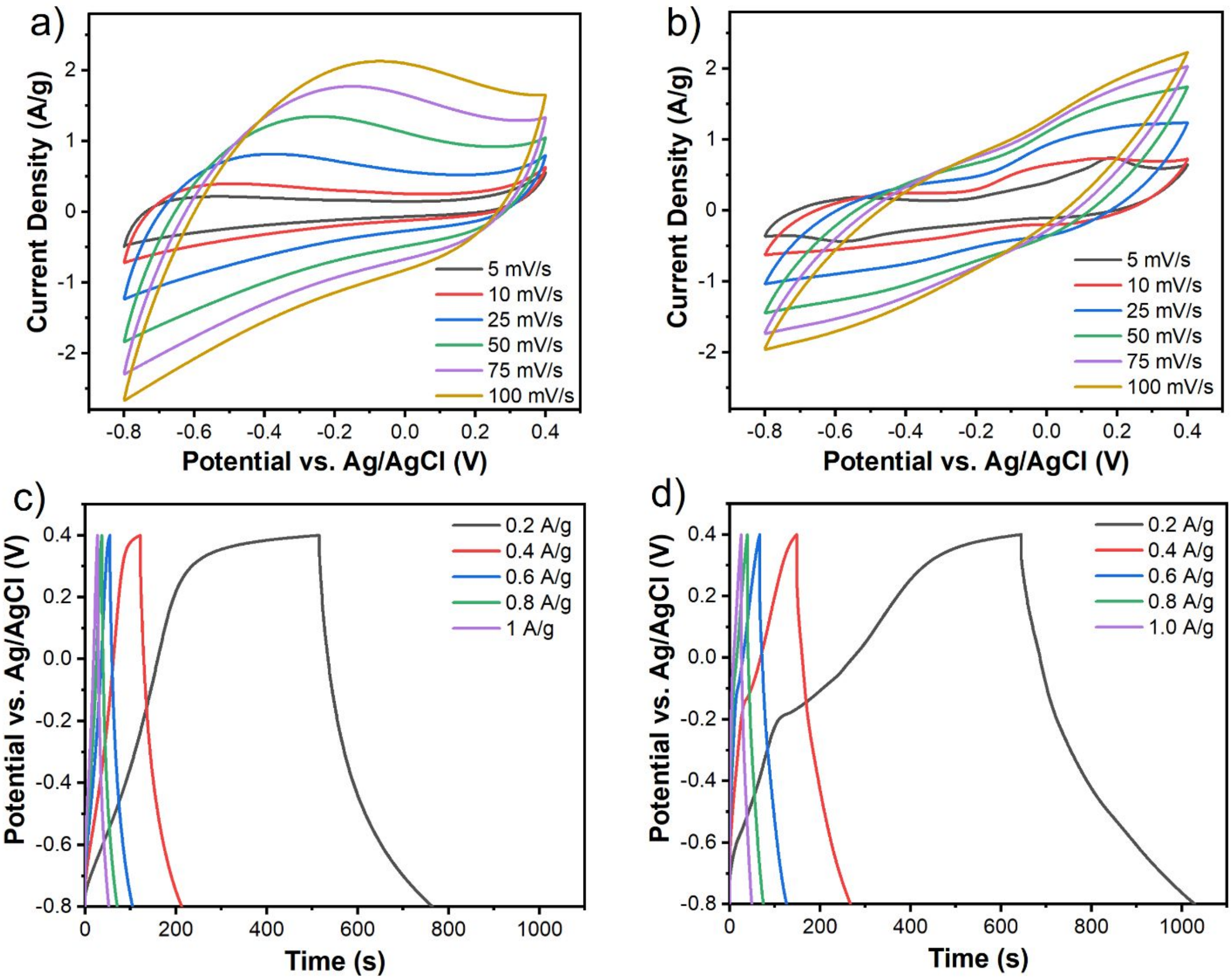

Figure S9. Electrochemical characterization of the structured carbon and carbon-cobalt composite in 3-electrode configuration. CV scans of the (a) structured carbon and (b) structured carbon-cobalt composite in the scan rate range of 5 to 100 mV/s. GCD curves at different current densities from 0.2 to 1.0 A/g for the (c) structured carbon and (d) structured carbon-cobalt composite.

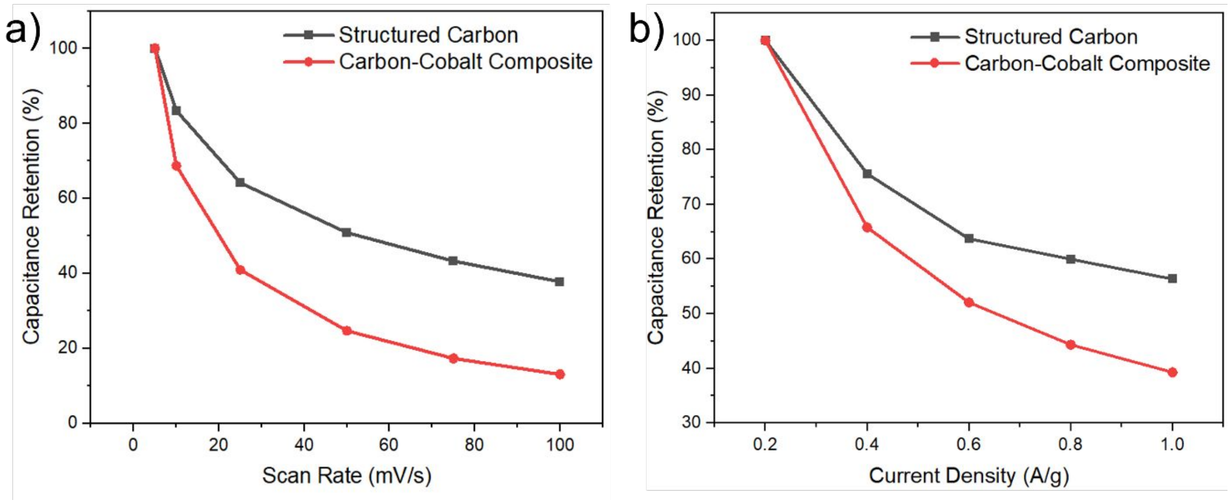

Figure S10. (a) Capacitance retention of the structured carbon and carbon-cobalt composite at different scan rates calculated from CV results. (b) Capacitance retention of the structured carbon and carbon-cobalt composite at different scan rates calculated from GCD results.

Table S1. Comparison of the electrochemical performance of our system with related literature reports on similar material systems.

Sample	Preparation method	Specific Capacitance	Reference
CoO@C array on Ni foam	Hydrothermal synthesis and CVD	3282.2 F/g @ 1mA/cm^2	1
LIC(MOF199@ZIF-67)	Direct laser scribing	5.02mF/cm^2 @ 0.2mA/cm^2	2
3D G aerogel/MnO_2	DIW, annealing, electrodeposition	18.74F/cm^2 @ 1mA/cm^2	3
PANI/rGO	DIW and chemical reduction	1329mF/cm^2 @ 4.2mA/cm^2	4
MoO_3/rGO	Inkjet printing and thermal reduction	22.5F/cm3 @ 1 A/cm^2	5
AC	SLA and carbonization	30F/g @0.01mV/s	6
$CoNi_2S_4$/NiCo-LDHs	SLA and electrodeposition	28.71F/cm^3 @ 10mA/cm^3	7
Structured carbon	SLS and carbonization	61F/g @ 5mV/s	This work
Structured carbon-cobalt composite	SLS and carbonization	92F/g @ 5mV/s	This work

References

(1) Wang, H.; Qing, C.; Guo, J.; A. Aref, A.; Sun, D.; Wang, B.; Tang, Y. Highly Conductive Carbon–CoO Hybrid Nanostructure Arrays with Enhanced Electrochemical Performance for

Asymmetric Supercapacitors. *J. Mater. Chem. A* **2014**, *2* (30), 11776–11783. https://doi.org/10.1039/C4TA01132E.

(2) Zhang, W.; Li, R.; Zheng, H.; Bao, J.; Tang, Y.; Zhou, K. Laser-Assisted Printing of Electrodes Using Metal–Organic Frameworks for Micro-Supercapacitors. *Adv. Funct. Mater.* **2021**, *31* (14), 2009057. https://doi.org/10.1002/adfm.202009057.

(3) Yao, B.; Chandrasekaran, S.; Zhang, J.; Xiao, W.; Qian, F.; Zhu, C.; Duoss, E. B.; Spadaccini, C. M.; Worsley, M. A.; Li, Y. Efficient 3D Printed Pseudocapacitive Electrodes with Ultrahigh MnO2 Loading. *Joule* **2019**, *3* (2), 459–470. https://doi.org/10.1016/j.joule.2018.09.020.

(4) Wang, Z.; Zhang, Q.; Long, S.; Luo, Y.; Yu, P.; Tan, Z.; Bai, J.; Qu, B.; Yang, Y.; Shi, J.; Zhou, H.; Xiao, Z.-Y.; Hong, W.; Bai, H. Three-Dimensional Printing of Polyaniline/Reduced Graphene Oxide Composite for High-Performance Planar Supercapacitor. *ACS Appl. Mater. Interfaces* **2018**, *10* (12), 10437–10444. https://doi.org/10.1021/acsami.7b19635.

(5) Li, B.; Hu, N.; Su, Y.; Yang, Z.; Shao, F.; Li, G.; Zhang, C.; Zhang, Y. Direct Inkjet Printing of Aqueous Inks to Flexible All-Solid-State Graphene Hybrid Micro-Supercapacitors. *ACS Appl. Mater. Interfaces* **2019**, *11* (49), 46044–46053. https://doi.org/10.1021/acsami.9b12225.

(6) Wang, P.; Zhang, H.; Wang, H.; Li, D.; Xuan, J.; Zhang, L. Hybrid Manufacturing of 3D Hierarchical Porous Carbons for Electrochemical Storage. *Adv. Mater. Technol.* **2020**, *5* (6), 1901030. https://doi.org/10.1002/admt.201901030.

(7) Chang, P.; Mei, H.; Tan, Y.; Zhao, Y.; Huang, W.; Cheng, L. A 3D-Printed Stretchable Structural Supercapacitor with Active Stretchability/Flexibility and Remarkable Volumetric Capacitance. *J. Mater. Chem. A* **2020**, *8* (27), 13646–13658. https://doi.org/10.1039/D0TA04460A.
